# Commentary: FSH receptor N680S genotype-guided gonadotropin choice increases cumulative pregnancy and live birth rates after *in vitro* fertilization

**DOI:** 10.3389/fendo.2025.1706120

**Published:** 2026-01-12

**Authors:** Livio Casarini, Manuela Simoni

**Affiliations:** 1 Unit of Endocrinology, Department of Biomedical, Metabolic and Neural Sciences, University of Modena and Reggio Emilia, Modena, Italy; 2 Center for Genomic Research-University of Modena and Reggio Emilia, Modena, Italy; 3 Unit of Endocrinology, Department of Medical Specialties, Azienda Ospedaliero-Universitaria di Modena-Baggiovara Hospital, Modena, Italy

**Keywords:** FSH, FSHR polymorphism, hMG, recombinant, urinary

## Introduction

This is a comment on the findings described by Hjelmér et al., 2025 (1) and their proposal to use urinary FSH (uFSH) preparations to improve assisted reproduction technique (ART) outcomes in women homozygous for the FSH receptor (FSHR) “680Ser” polymorphic variant (rs6166). We aimed to appraise methodological flaws and interpretative issues in the study by Hjelmér et al. Relevant concerns arise from the interpretation of the results and their use to support the conclusion that uFSH should be recommended to treat genotyped FSHR 680Ser carriers.

## Inaccurate terminology leads to misinterpretation of results

The commercial preparation Menopur (Ferring Pharmaceuticals SA, Saint-Prex, Switzerland), improperly defined by the authors as a uFSH, is a mixture of FSH and LH obtained from menopausal women, with the addition of hCG (2), and should be indicated as “menopausal gonadotropins” (hMG). uFSH drugs contain exclusively FSH molecules purified from urine, which is not the case for Menopur (3). Although this may appear to be a semantic issue, inaccurate terminology may have led to the misinterpretation that FSH of urinary origin elicits an *in vivo* response that could be, at least in part, attributable to LH and hCG. This may underlie some of the result misinterpretations discussed below.

## *In vitro* data do not support authors’ conclusion

In their study, Hjelmér and colleagues performed *in vitro* experiments intended to support their clinical observations. They measured cyclic adenosine monophosphate (cAMP) production in transfected COS-1 cells treated with recombinant FSH (rFSH) and hMG. However, these cells express only FSHR, preventing the action of LH/hCG molecules, which exert their effects through the LH/hCG receptor (LHCGR). Therefore, this model is not representative of clinical ovarian stimulation, in which granulosa cells express both FSHR and LHCGR, and theca cells express LHCGR (4). LH/hCG are present in Menopur but absent in the rFSH reference drug (Gonal-F, Merck KGaA, Darmstadt, Germany) (2). The efficacy of FSH and hCG varies considerably depending on whether LHCGR is co-expressed with FSHR [see Table 2 in ref (2)]. However, the *in vitro* system used by Hjelmér et al. does not allow detection of the contribution of LH/hCG to cAMP activation because of the lack of LHCGR expression.

Once it is established that the cellular response shown in Figure 2 (1) is mediated by FSH molecules only, it is not surprising that cAMP levels differ between the hMG and rFSH groups in FSHR-expressing COS-1 cells. These considerations raise concerns about the actual FSH concentration administered *in vitro* and how these doses were determined. Specifically, the authors used international units (IU) rather than molarity, introducing a bias: IU is not a measure of hormone quantity, as it reflects *in vivo* activity in rodents (5), whereas molarity is more appropriate for *in vitro* experiments because it reflects the number of molecules. There is no direct proportionality between Menopur and Gonal-F IU values, as they are based on the activity of different molecules [Table 2 in ref. (2)]. By using IU *in vitro*, the authors applied different concentrations of FSH, with a clear overdosing in the Menopur-treated group. In addition, no information is provided on the stimulation volume, making it impossible to determine the actual hormone concentration used, while the reported quantity (up to 90 IU) appears extremely high for *in vitro* experiments.

*The in vitro* data also present methodological and statistical issues, as a t-test was inappropriately used to analyze multiple groups. The authors compared three groups (treatments: “uFSH,” “rFSH,” and “no FSH”) across two variables (FSH dose: 0, 1, 10, and 90 IU). A two-way ANOVA, or a *non*-parametric equivalent such as the Kruskal–Wallis test, should have been used instead of a t-test, which is limited to two-group comparisons and may lead to type I errors when applied to multiple testing (6). Finally, the number of independent replicates and the buffer volume used for cell treatment are not reported, and no data are shown for COS-1 cells expressing FSHR 680Asn or FSHR 680Asn/680Ser.

In summary, insufficient information is provided to allow the *in vitro* experiments to be reproduced or correctly interpreted. Therefore, these results do not support the authors’ conclusions.

## Major flaws of the clinical study

The main problem is the lack of a clinical study registration number, which prevents evaluation of whether the experimental design was modified *a posteriori* and biased by data selection, potentially performed after the initial analyses did not reveal any difference. This observation is particularly relevant given the retrospective nature of the study (1).

Other key information is missing. For instance, the drug used to treat *non*-genotyped women is not indicated, preventing its use as a control group. Results may be biased by gonadotropin dosing, which is higher in the hMG group [see Table 1 in the paper (1)]. Details on how adjustments for BMI, age, and fertilization method were performed are also missing. Other relevant data impacting ART prognosis are not reported, such as antral follicle count (AFC) (7). In summary, the study design is not appropriate for testing the authors’ hypothesis.

## Opposite data

The authors did not reconcile their results with the existing literature.

There is no clear evidence demonstrating that uFSH elicits different responses than rFSH, weakening the rationale of the study by Hjelmér et al. Commercial FSH preparations are mixtures of different glycoforms of the same molecule, but they rarely trigger clear preparation-specific responses due to the lack of substantial diversity in overall composition and half-life among these mixtures (3, 8, 9). Many clinical studies support or refute the superiority of rFSH vs uFSH, and vice versa (10–15). However, they have not produced converging evidence for the superiority of one drug in improving clinical outcomes (14). Instead, previous findings consistently support that differences between rFSH and hMG are due to LH/hCG (16), as these ligands modulate the response to FSH through LHCGR activation (17), regardless of FSHR genotype. This concept was demonstrated in a large *in vitro* dataset (18), in which human primary granulosa lutein cells were treated with rFSH in the presence and absence of recombinant LH (Luveris, Merck KGaA). LH improved cellular responses regardless of FSHR genotype, suggesting that LH/hCG addition may have an indication in poor/sub-responder patients, rather than in a subgroup of FSHR 680Ser carriers (18) (see Supplementary Tables S3, S4). Rigorous meta-analysis (19) and a registry-based study (20) consolidated this view, showing that LHCGR ligands may be used to personalize FSH treatments when the ovarian response to FSH needs to be optimized.

In conclusion, the study by Hjelmér et al. fails to provide indications for the use of hMG—and even less so uFSH—based on FSHR polymorphism. Improper study design, missing key information, lack of support from *in vitro* experiments, and data misinterpretations undermine the authors’ conclusions. Converging data suggest that LH/hCG addition to FSH may benefit ART patients with poor/sub-response to FSH, regardless of FSHR genotype.

## References

[1] Hjelmér I Nilsson M Henic E Jędrzejczak P Nenonen H Ozegowska K . FSH receptor N680S genotype-guided gonadotropin choice increases cumulative pregnancy and live birth rates after *in vitro* fertilization. Front Endocrinol (Lausanne). (2025) 16:1576090. doi: 10.3389/FENDO.2025.1576090, PMID: 40433406 PMC12106330

[2] Casarini L Riccetti L Paradiso E Benevelli R Lazzaretti C Sperduti S . Two human menopausal gonadotrophin (hMG) preparations display different early signaling in *vitro*. Mol Hum Reprod. (2020) 26:894–905. doi: 10.1093/MOLEHR/GAAA070, PMID: 33084890

[3] Riccetti L Sperduti S Lazzaretti C Klett D De Pascali F Paradiso E . Glycosylation pattern and *in vitro* bioactivity of reference follitropin alfa and biosimilars. Front Endocrinol (Lausanne). (2019) 10:503. doi: 10.3389/fendo.2019.00503, PMID: 31396162 PMC6667556

[4] Liu YX Hsueh AJW . Synergism between granulosa and theca-interstitial cells in estrogen biosynthesis by gonadotropin-treated rat ovaries: Studies on the two-cell, two-gonadotropin hypothesis using steroid antisera. Biol Reprod. (1986) 35:27–36. doi: 10.1095/biolreprod35.1.27, PMID: 3091103

[5] Steelman SL Pohley FM . Assay of the follicle stimulating hormone based on the augmentation with human chorionic gonadotropin. Endocrinology. (1953) 53:604–16. doi: 10.1210/endo-53-6-604, PMID: 13116950

[6] Mitchell PJ . Experimental Design and Statistical Analysis for Pharmacology and the Biomedical Sciences. John Wiley & Sons, Inc (2022). doi: 10.1002/9781119437642

[7] Alviggi C Andersen CY Buehler K Conforti A De Placido G Esteves SC . A new more detailed stratification of low responders to ovarian stimulation: from a poor ovarian response to a low prognosis concept. Fertil Steril. (2016) 105:1452–3. doi: 10.1016/J.FERTNSTERT.2016.02.005, PMID: 26921622

[8] Le Cotonnec JY Porchet HC Beltrami V Khan A Toon S Rowland M . Clinical pharmacology of recombinant human follicle-stimulating hormone (FSH). I. Comparative pharmacokinetics with urinary human FSH. Fertil Steril. (1994) 61:669–78. doi: 10.1016/S0015-0282(16)56644-8, PMID: 8150109

[9] Karlsson MO Wade JR Loumaye E Munafo A . The population pharmacokinetics of recombinant- and urinary-human follicle stimulating hormone in women. Br J Clin Pharmacol. (1998) 45:13–20. doi: 10.1046/J.1365-2125.1998.00644.X, PMID: 9489588 PMC1873998

[10] Matorras R Osuna C Exposito A Crisol L Pijoan JI . Recombinant FSH versus highly purified FSH in intrauterine insemination: Systematic review and metaanalysis. Fertil Steril. (2011) 95:1937–42. doi: 10.1016/j.fertnstert.2011.02.030, PMID: 21429486

[11] Abd-Elaziz K Duijkers I Stöckl L Dietrich B Klipping C Eckert K . A new fully human recombinant FSH (follitropin epsilon): Two phase i randomized placebo and comparator-controlled pharmacokinetic and pharmacodynamic trials. Hum Reprod. (2017) 32:1639–47. doi: 10.1093/humrep/dex220, PMID: 28591833

[12] Lledó B Dapena P Ortiz JA Morales R Llacer J Bernabeu R . Clinical efficacy of recombinant versus highly purified follicle-stimulating hormone according to follicle-stimulating hormone receptor genotype. Pharmacogenet Genomics. (2016) 26:288–93. doi: 10.1097/FPC.0000000000000215, PMID: 26959715

[13] Bergandi L Canosa S Carosso AR Paschero C Gennarelli G Silvagno F . Human recombinant FSH and its biosimilars: Clinical efficacy, safety, and cost-effectiveness in controlled ovarian stimulation for *in vitro* fertilization. Pharmaceuticals. (2020) 13:1–23. doi: 10.3390/ph13070136, PMID: 32605133 PMC7407829

[14] Lunenfeld B Bilger W Longobardi S Alam V D’Hooghe T Sunkara SK . The development of gonadotropins for clinical use in the treatment of infertility. Front Endocrinol (Lausanne). (2019) 10:429. doi: 10.3389/fendo.2019.00429, PMID: 31333582 PMC6616070

[15] Liu X Hao C Wang J . Efficacy of highly purified urinary FSH versus recombinant FSH in Chinese women over 37 years undergoing assisted reproductive techniques. Int J Fertil Steril. (2015) 8:385–92. doi: 10.22074/ijfs.2015.4178, PMID: 25780520 PMC4355925

[16] Bosch E Alamá P Romero JL Marí M Labarta E Pellicer A . Serum progesterone is lower in ovarian stimulation with highly purified HMG compared to recombinant FSH owing to a different regulation of follicular steroidogenesis: A randomized controlled trial. Hum Reprod. (2024) 39:393–402. doi: 10.1093/humrep/dead251, PMID: 38037188

[17] Casarini L Riccetti L De Pascali F Nicoli A Tagliavini S Trenti T . Follicle-stimulating hormone potentiates the steroidogenic activity of chorionic gonadotropin and the anti-apoptotic activity of luteinizing hormone in human granulosa-lutein cells. vitro. Mol Cell Endocrinol. (2016) 422:103–14. doi: 10.1016/J.MCE.2015.12.008, PMID: 26690776

[18] Sperduti S Paradiso E Anzivino C Lazzaretti C Limoncella S D’Alessandro S . LH increases the response to FSH in granulosa-lutein cells from sub/poor-responder patients. vitro. Hum Reprod. (2023) 38:103–12. doi: 10.1093/HUMREP/DEAC246, PMID: 36367827

[19] Santi D Casarini L Alviggi C Simoni M . Efficacy of follicle-stimulating hormone (FSH) alone, FSH + Luteinizing hormone, human menopausal gonadotropin or FSH + Human chorionic gonadotropin on assisted reproductive technology outcomes in the “Personalized” Medicine era: A meta-analysis. Front Endocrinol (Lausanne). (2017) 8:114. doi: 10.3389/FENDO.2017.00114, PMID: 28620352 PMC5451514

[20] Arvis P Massin N Lehert P . Effect of recombinant LH supplementation on cumulative live birth rate compared with FSH alone in poor ovarian responders: a large, real-world study. Reprod BioMed Online. (2021) 42:546–54. doi: 10.1016/J.RBMO.2020.08.035, PMID: 33431337

